# 应用组织芯片法检测EGFR、COX-2在肺癌中的表达及生物学意义

**DOI:** 10.3779/j.issn.1009-3419.2010.02.05

**Published:** 2010-02-20

**Authors:** 丛中 朱, 娟 刘, 新允 王

**Affiliations:** 300070 天津，天津医科大学病理学教研室 Department of Pathology, Tianjin Medical University, Tianjin 300070, China

**Keywords:** 肺肿瘤, EGFR, COX-2, Lung neoplasms, Epidermal growth factor receptor, Cyclooxygenase-2

## Abstract

**背景与目的:**

研究表明表皮生长因子受体(epidermal growth factor receptor, EGFR)和环氧合酶-2 (cyclooxygenase-2, COX-2)在多种实体瘤中存在高表达, 并且可以通过相应的信号通路调节肿瘤的生长、侵袭和转移。本研究旨在探讨EGFR和COX-2在人类肺癌组织中表达的生物学意义及相互之间的关系。

**方法:**

应用组织芯片技术结合免疫组织化学SP法检测89例原发肺癌、12例淋巴结转移性肺癌、12例癌前病变(不典型腺瘤样增生)和10例正常肺组织中EGFR、COX-2蛋白的表达情况。

**结果:**

EGFR在肺癌组、癌前病变组、淋巴结转移性肺癌组中的阳性表达率分别为59.6%(53/89)、41.7%(5/12)和66.7%(8/12), COX-2在上述三组中的阳性表达率分别为52.8%(47/89)、41.7%(5/12)和66.7%(8/12), 均较正常组明显升高(*P* < 0.05)。EGFR和COX-2的表达与肺癌的组织学类型、临床分期和淋巴结转移有关(*P* < 0.05), 而与组织学分级、性别、年龄无关(*P* > 0.05)。COX-2的表达还与肺癌的大体类型有关(*P* < 0.05)。EGFR和COX-2的表达呈正相关(*P* < 0.01)。

**结论:**

EGFR和COX-2在肺癌中的异常表达与肺癌的发生、发展和恶性程度有关, 两者存在一定的协同作用。检测EGFR和COX-2的表达有助于肺癌的临床诊断和预后评估。

## Abstract (en)

**Background and objective:**

Epidermal growth factor receptor (EGFR) and cyclooxygenase-2 (COX-2), which can regulate growth, invasion and metastasis of tumor through relevant signaling pathway, have been detected in a variety of solid tumors.The aim of this study is to investigate the biological significance of EGFR and COX-2 expression in lung cancer and the relationship between them.

**Methods:**

The expression of EGFR and COX-2 was detected in 89 primary lung cancer tissues, 12 premaliganant lesions, 12 lymph node metastases, and 10 normal lung tissues as the control by immunohistochemical method on a tissue microarray section.

**Results:**

EGFR protein was detectable in 59.6%, 41.7%, and 66.7% of primary lung cancer tissues, premalignant lesions and lymph node metastases, respectively; COX-2 protein was detectable in 52.8%, 41.7%, and 66.7% of primary lung cancer tissues, premalignant lesions and lymph node metastases, respectively, which were significantly higher than those of the control (*P* < 0.05).The positive ratios and the levels of the expression of EGFR and COX-2 proteins were closely related to histological type, clinical stage and lymph node metastasis of lung cancer (*P* < 0.05), but not to histological grade, sex and age (*P* > 0.05).COX-2 expression was related to gross type (*P* < 0.05).A highly positive correlation was observed between EGFR and COX-2 expression (*P* < 0.01).

**Conclusion:**

Overexpression of EGFR and COX-2 may play an important role in the tumorgenesis, progression and malignancy of lung cancer.Detection of EGFR and COX-2 expression might be helpful to diagnosis and prognosis of lung cancer.

肺癌是一种严重威胁人类健康与生命安全的疾病, 其发病率和死亡率呈逐年上升的趋势。表明表皮生长因子受体(epidermal growth factor receptor, EGFR)在大多数实体肿瘤中高表达, 可以促进肿瘤细胞的增殖、侵袭、转移以及肿瘤血管生成^[1]^。环氧合酶-2 (cyclooxygenase-2, COX-2)在多种癌前病变、恶性肿瘤和转移癌中的表达均异常升高, 并且升高的水平与肿瘤的生长、侵袭和预后有关^[2]^。检测两者在肺癌中的表达及相关性, 并探讨它们与肺癌各临床参数之间的关系, 对肺癌的诊断、预后判断和治疗具有重要意义。

## 1 材料和方法

### 1.1 材料

#### 1.1.1 病例与标本

收集天津医科大学总医院及第二附属医院病理科1987年-2003年手术切除的肺癌石蜡包埋标本, 其中原发肺癌89例, 淋巴结转移性肺癌12例, 肺癌癌前病变12例。患者术前均未行放疗及化疗。原发肺癌中包括腺癌35例, 鳞癌33例, 小细胞癌12例, 大细胞癌9例; 男性67例, 女性22例; 年龄33岁-78岁, 平均年龄60.28±9.48岁; 周围型肺癌59例, 中央型肺癌30例; 高-中分化53例, 低-未分化36例; Ⅰ+Ⅱ期56例, Ⅲ+Ⅳ期33例; 有淋巴结转移者48例, 无淋巴结转移者41例。收集10例正常肺组织作为对照组。

#### 1.1.2 试剂

兔抗人EGFR单克隆抗体(ZA-0505, 工作液)、兔抗人COX-2单克隆抗体(ZA-0515, 工作浓度1:80)、SP-9000试剂盒(包括封闭用山羊血清, 生物素标记二抗和辣根酶标记链酶卵白素)和DAB显色试剂盒购自北京中杉生物技术有限公司。

### 1.2 方法

#### 1.2.1 组织芯片制作

由于组织芯片技术是将数十或百个组织片排列于一张切片上, 可以同时研究细胞形态、特定基因蛋白在样本中的表达和功能状态, 并且实验一次完成, 实验条件一致, 大大降低了实验误差, 增加了结果的可比性和可重复性, 特别适用于临床病理研究中, 因此本次研究设计采用了该方法。制作方法如下:选取存档蜡块, 切片, HE染色, 病理专家复诊, 对蜡块定位。美国Beecher阵列仪在受体蜡块上按阵列图打孔, 组织芯直径为0.6 mm, 原发性肺癌和正常肺组织每例取2芯, 淋巴结转移性肺癌和癌前病变每例取3芯。用穿刺针将标记好的供体组织芯取出, 转移到受体蜡块相应位置制成组织芯片蜡块。采用3 μm切片^[3]^, 切片后组织芯均匀排布于载玻片上, 无脱片, 也无明显移位及扭曲。

#### 1.2.2 免疫组化SP法

切片脱蜡水化后, 3%H_2_O_2_封闭内源性过氧化物酶10 min, 0.1%枸橼酸缓冲液(pH6.0-6.2)中微波修复20 min, 山羊血清封闭15 min, 滴加一抗, 4 ℃冰箱过夜。第二天切片恢复室温后, 依次加入生物素化的二抗和辣根酶标记链酶卵白素, 分别37 ℃下孵育30 min。DAB显色, 苏木素复染, 梯度酒精脱水, 二甲苯透明, 中性树胶封片。滴加PBS取代一抗作为阴性对照。

#### 1.2.3 结果判定

EGFR阳性表达位于细胞膜和细胞浆(图 1), COX-2定位于细胞浆(图 2)。根据免疫组化染色强度及阳性细胞数量进行半定量评分^[4]^, 其中染色强度以多数细胞着色深浅计算:不着色为0分, 浅棕黄色为1分, 棕黄色为2分, 深棕黄色为3分; 阳性细胞数 < 10%为0分, 10%-45%为1分, 46%-65%为2分, > 65%为3分; 染色强度及阳性细胞数得分相乘, 0为阴性(-), 1分-2分为弱阳性(+), 3分-4分为阳性(++), 5分-6分为强阳性(+++)。

**1:**
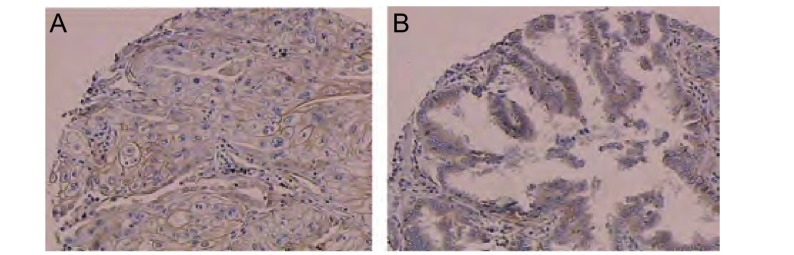
EGFR在肺癌组织中的表达(SP, ×200) The expression of EGFR protein in lung cancers (SP, ×200)

**2:**
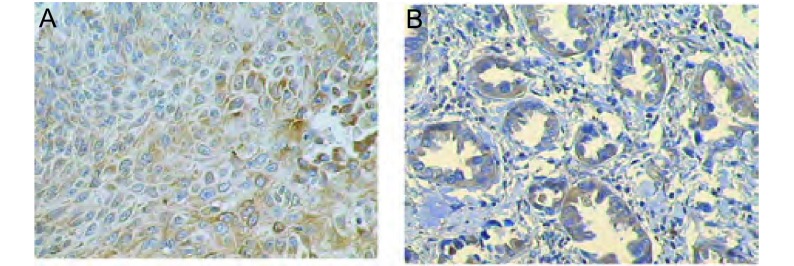
COX-2在肺癌组织中的表达(SP, ×400) The expression of COX-2 protein in lung cancers (SP, ×400)

### 1.3 统计学分析

采用SPSS 13.0统计软件包进行数据分析。各组阳性率的比较用*χ*^2^检验或*Fisher*确切概率法, 各指标表达水平的比较采用非参数检验(*Mann-Whitney*检验或*Kruskal-Wallis*检验), EGFR和COX-2表达的相关性分析采用*Spearman*等级相关, *P* < 0.05为差异有统计学意义。

## 2 结果

### 2.1 EGFR的表达情况

EGFR在各组的阳性表达率为原发性肺癌组59.6%(53/89), 癌前病变组41.7%(5/12), 淋巴结转移性肺癌组66.7%(8/12), 正常肺组织组为10.0%(1/10), 四组间EGFR的阳性表达率有统计学差异(*P* < 0.05), 淋巴结转移性肺癌组 > 原发性肺癌组 > 癌前病变组 > 正常组。EGFR的表达在肺癌的组织学类型、临床分期和有无淋巴结转移各组间有统计学差异(*P* < 0.01), 其中大细胞癌 > 腺癌 > 鳞癌 > 小细胞癌, 非小细胞癌 > 小细胞癌, 临床Ⅲ+Ⅳ期 > Ⅰ+Ⅱ期, 有淋巴结转移组高于无淋巴结转移组。EGFR的表达强度随临床分期的增加有上升的趋势, 合并分期后Ⅲ+Ⅳ期高于Ⅰ+Ⅱ期(*U*=474.500, *P* < 0.001)。EGFR的阳性表达率与肺癌的大体类型、组织学分级、性别和年龄无关(*P* > 0.05)。见表 1。

**1:** EGFR和COX-2的表达及与临床病理学参数的关系 Associations between expression of EGFR and COX-2 and clinicopathologic parameters

Variables and Categories	*n*	Expression of EGFR		*χ*^2^	*P*	Expression of COX-2		*χ*^2^	*P*
		Positive	Positive rate			Positive	Positive rate		
Samples				10.413	0.015			11.881	0.008
Primary lung cancer	89	53	59.60%			47	52.80%		
Premalignant lesion	12	5	41.70%			5	41.70%		
Metastatic lung cancer in lymph node	12	8	66.70%			8	66.70%		
Normal lung tissue	10	1	10.00%			0	0		
Sex				0.203	0.653			0.035	0.851
Male	67	39	58.20%			35	52.20%		
Female	22	14	63.60%			12	54.50%		
Age				0.037	0.847			0.191	0.662
> 60	53	32	60.40%			29	54.70%		
< 60	36	21	58.30%			18	50.00%		
Gross type				0.726	0.394			15.777	< 0.001
Peripheral type	59	37	62.70%			40	67.80%		
Central type	30	16	53.30%			7	23.30%		
Histologic type				12.945	0.005			18.896	< 0.001
Adenocarcinoma	35	23	65.70%			21	60.00%		
Squamous cell carcinoma	33	20	60.60%			18	54.50%		
Large cell lung cancer	9	8	88.90%			8	88.90%		
Small cell lung caner	12	2	16.70%			0	0		
Non-small cell lung cancer	77	51	66.20%	- 0.003^*^	47	61.00%	15.521	< 0.001^*^
Differentiation				0.037	0.847			0.001	0.996
High-moderate	53	32	60.40%			28	52.80%		
Low-undifferentiation	36	21	58.30%			19	52.80%		
Stage				8.058	0.005			4.041	0.044
Ⅰ+Ⅱ	56	27	48.20%			25	44.60%		
Ⅲ+Ⅳ	33	26	78.80%			22	66.70%		
Lymph node metastasis				10.325	0.001			3.927	0.048
Present	48	36	75%			30	62.50%		
Absent	41	17	41.50%			16	41.50%		
^*^non-small cell lung cancer *vs* small cell lung caner.									

### 2.2 COX-2的表达情况

COX-2的阳性表达率为原发性肺癌组52.8%(47/89), 癌前病变组41.7%(5/12), 淋巴结转移性肺癌66.7%(8/12), 正常组为0(0/10), 淋巴结转移性肺癌组 > 原发性肺癌组 > 癌前病变组 > 正常组, 四组间的COX-2的阳性表达率有统计学差异(*P* < 0.01)。COX-2的表达在肺癌的大体类型、组织学类型、临床分期和有无淋巴结转移各组间有统计学差异(*P* < 0.05), 其中周围型肺癌 > 中央型肺癌, 大细胞癌 > 腺癌 > 鳞癌 > 小细胞癌, 非小细胞癌 > 小细胞癌, 临床Ⅲ+Ⅳ期 > Ⅰ+Ⅱ期, 有淋巴结转移组高于无淋巴结转移组。COX-2的表达强度随临床分期的增加有上升的趋势, 合并分期后Ⅲ+Ⅳ期高于Ⅰ+Ⅱ期(*U*=680.500, *P*=0.026)。COX-2的阳性表达率与肺癌的组织学分级、性别和年龄无关(*P* > 0.05)。见表 1。

### 2.3 EGFR和COX-2表达的相关性

EGFR与COX-2在肺癌组织中的表达成正相关(*r_s_*=0.495, *P* < 0.01)。

## 3 讨论

EGFR是ErbB受体酪氨酸激酶家族的成员之一, 它是一个分子量为170 kDa的单跨膜酪氨酸激酶, 其编码基因位于染色体7p13-q22, 通过与相应配体如表皮生长因子(epidermal growth factor, EGF)或转化生长因子-α(transforming growth factor-α, TGF-α)结合, EGFR被活化, 其与自身分子发生同二聚体化或与同家族的其它分子发生异二聚体化, 导致EGFR胞浆部分的特定酪氨酸残基磷酸化, 并刺激下游信号传导通路的活化, 从而调控细胞的增殖、转移、黏附、分化以及存活^[5, 6]^。

文献报道, 在膀胱癌^[7]^、骨肉瘤^[8]^、胆道肿瘤^[9]^等多种实体瘤组织中存在*EGFR*基因的扩增或蛋白的高表达。本研究中EGFR在淋巴结转移性肺癌、原发性肺癌和癌前病变中高表达, 在正常肺组织中低表达, 提示EGFR与肿瘤的发生关系密切, 其异常表达可能是肺癌发生的关键因素之一。最近的研究^[10]^表明肺腺癌中*EGFR*基因拷贝数增加和EGFR蛋白的过表达, 此结果与我们的研究结果相符。因此检测EGFR在肺癌中的表达情况对肺癌的临床诊断有重要的指导作用。我们还发现EGFR的表达与肺癌的组织学类型密切相关, 大细胞癌 > 腺癌 > 鳞癌 > 小细胞癌, 非小细胞癌高于小细胞癌, 提示EGFR在肺癌中的过表达存在组织学特异性, 不同组织学类型肺癌的发病机理可能不同, 因而治疗手段和方案也各异, 这就为肺癌的诊断和针对性治疗提供了一定的依据。

本研究结果显示晚期肺癌患者EGFR的阳性表达率高于早期患者, 并且随着临床分期的增加EGFR水平增强。EGFR表达还与肺癌淋巴结转移密切相关, 有淋巴结转移患者的表达率高于无淋巴结转移者。这些发现均提示EGFR与肺癌的侵袭和转移密切相关。Kim等^[11]^报道EGFR是估计胃癌预后的独立指标, 过表达者主要为晚期患者, 其缓解期和生存期都明显短于阴性表达者。我们推测EGFR它可能通过增强瘤细胞增殖能力、调控肿瘤细胞的粘附、肿瘤性血管生成而促进肺癌转移, 对肺癌患者进行EGFR表达的检测将有助于患者的预后评估。

COX-2是花生四烯酸合成前列腺素过程中的一个重要限素酶, 研究^[2]^表明它与肿瘤的发生、进展、侵袭和血管生成密切相关。本研究显示, COX-2在癌前病变组、原发性肺癌组和淋巴结转移性肺癌组中表达率依次升高, 并明显高于正常肺组织, 提示COX-2可促进肺癌的发生和进展。尤其在非小细胞肺癌中COX-2呈高表达, 小细胞肺癌中表达阴性, 提示COX-2在非小细胞肺癌发生发展中起重要作用。本研究结果还显示周围型肺癌中COX-2的表达水平明显高于中央型肺癌组, 其原因可能是周围型肺癌多为大细胞癌和腺癌, 中央型肺癌多为鳞癌。我们的结果也证实大细胞癌 > 腺癌 > 鳞癌, 两结果彼此相符。因此从肉眼类型也反映了不同类型肺癌中COX-2的作用不同, 为不同类型肺癌的临床诊断和治疗提供了思路。本研究结果还显示晚期患者以及伴淋巴结转移患者COX-2阳性表达率升高, 并且COX-2的表达强度随临床分期的增加有上升的趋势。文献^[12, 13]^报道COX-2的表达与肿瘤的分级、高增殖率、低凋亡指数有关, 高表达患者预后较差。我们研究的结果和文献报道一致, 提示COX-2与肺癌的临床进展密切相关, 可作为预测预后的重要指标, 临床检测COX-2的表达有助于患者治疗方案的选择和预后评估。

COX-2和EGFR的激活与调节均与丝裂原活化蛋白激酶(mitogen-activated protein kinase, MAPK)和磷脂酰肌醇3-激酶(phosphatidylinositol 3-kinase, PI3K)等信号通路有关, 通过这些信号通路COX-2和EGFR之间的关系似乎就建立起来。迄今为止, 国内外许多研究者^[1, 14]^对两者之间的关系作了一系列研究, 发现COX-2和EGFR之间可能存在复杂的相互调控关系, COX-2可以被EGFR活化而激活, COX-2表达的增加可以增加前列腺素E2(prostaglandin E2, PGE2)的产生, 而PGE2可以转而活化EGFR。Vallbohmer等^[15]^的研究结果证实, COX-2、EGFR和白细胞介素-8(interleukin-8, IL-8)的表达与西妥昔单抗(cetuximab, 爱必妥)单一治疗后患者总的生存率有关, 三者低表达的患者存活时间明显长于高表达者。我们通过对COX-2和EGFR进行检测, 发现两者的表达呈正相关, 结果与文献报道一致。本研究结果显示COX-2和EGFR均可以调节肺癌的发生和发展, 并且两者之间存在相互调控作用。因此, COX-2和EGFR在肺癌发生、发展、浸润和转移过程中起重要作用, 检测EGFR和COX-2的表达有助于肺癌的临床诊断和预后评估。
